# Causal effects of 731 immune cell phenotypes on autism spectrum disorder: a Mendelian randomization study

**DOI:** 10.3389/fpsyt.2024.1397006

**Published:** 2024-05-16

**Authors:** Yunfeng Yu, Xinyu Yang, Gang Hu, Yuman Yin, Rong Yu

**Affiliations:** ^1^School of Traditional Chinese Medicine, Hunan University of Chinese Medicine, Changsha, Hunan, China; ^2^Department of Endocrinology, The First Hospital of Hunan University of Chinese Medicine, Changsha, Hunan, China

**Keywords:** immune cell, phenotype, autism spectrum disorder, genome-wide association study, Mendelian randomization

## Abstract

**Objective:**

The role of different immune cells in autism spectrum disorders (ASD) is still controversial. The purpose of this study was to evaluate the causal effects of different immune cell phenotypes on ASD via Mendelian randomization (MR).

**Methods:**

Datasets of immune cell phenotypes were obtained from the European Bioinformatics Institute, and datasets of ASD were obtained from the IEU Open GWAS project. Single nucleotide polymorphisms were selected based on the assumptions of association, independence, and exclusivity. Inverse variance weighted was utilized as the main method for MR analysis. MR-Egger was employed to assess the horizontal pleiotropy of the results. Cochran’s Q and leave-one-out method were used for heterogeneity analysis and sensitivity analysis of the results, respectively.

**Results:**

MR analysis showed that TD CD8br AC [odds ratio (OR), 1.137; 95% confidence interval (CI), 1.031–1.254; *p* = 0.010], CD8br %leukocyte (OR, 1.142; 95% CI, 1.067–1.223; *p* < 0.001), CD8br and CD8dim %leukocyte (OR, 1.117; 95% CI, 1.032–1.210; *p* = 0.006), naive CD8br %T cell (OR, 1.052; 95% CI, 1.004–1.104; *p* = 0.035), CD28− CD8dim %T cell (OR, 1.097; 95% CI, 1.038–1.158; *p* < 0.001), CD127− CD8br AC (OR, 1.086; 95% CI, 1.006–1.171; *p* = 0.034), CD45 on CD8br (OR, 1.059; 95% CI, 1.021–1.099; *p* = 0.002), CD3 on HLA DR+ CD8br (OR, 1.098; 95% CI, 1.041–1.158; *p* < 0.001), CD4 on activated Treg (OR, 1.048; 95% CI, 1.001–1.096; *p* = 0.046), CD3 on CD39+ resting Treg (OR, 1.070; 95% CI, 1.012–1.131; *p* = 0.018), IgD+ CD38− %lymphocyte (OR, 1.103; 95% CI, 1.023–1.190; *p* = 0.011), CD62L− plasmacytoid DC %DC (OR, 1.046; 95% CI, 1.001–1.093; *p* = 0.046), and FSC-A on plasmacytoid DC (OR, 1.075; 95% CI, 1.003–1.153; *p* = 0.042) were associated with increased genetic susceptibility to ASD. MR-Egger displayed no horizontal pleiotropy (*p* ≥ 0.05). Cochran’s Q revealed no heterogeneity of results (*p* ≥ 0.05). Sensitivity analysis indicated that the results were robust.

**Conclusion:**

This MR analysis revealed 13 immune cell phenotypes associated with increased genetic susceptibility to ASD and emphasized the importance of CD8 T cells and Tregs, which provides new directions for the pathogenesis and drug research of ASD.

## 1 Introduction

Autism spectrum disorder (ASD) is a heterogeneous neurodevelopmental disorder characterized by social communication and interaction deficits, restricted interests, and repetitive behaviors (1). An epidemiologic study showed that the global prevalence of ASD is approximately 0.6% (2). With the increasing number of affected individuals, the public health impact of ASD is becoming more prominent (3). It is reported that the annual economic cost of ASD in the United States reached $268 billion in 2015 and is expected to grow to $461 billion by 2025 (4). Despite substantial research funding globally dedicated to ASD-related studies, there is still a lack of specific therapeutic drugs targeting the core features of ASD (5). The pathogenesis of ASD has not yet been fully elucidated. Previous views suggested that it is related to factors such as genetic factors, metabolic disorders, mitochondrial dysfunction, and oxidative stress (6). As research progresses, more and more researchers are recognizing that immune system dysfunction may play an essential role in the pathogenesis of ASD (7). Therefore, understanding the role of immune cells in the pathogenesis or progression of ASD aids in developing cutting-edge diagnostic and therapeutic strategies.

Immune cells, including T cells, B cells, and monocytes, may be involved in the progression of ASD (8). Compared to the healthy population, peripheral blood CD4+ T lymphocytes and Tregs cells are significantly reduced and Th17 cells are significantly increased in ASD patients (9). Further studies have shown that children with ASD have a higher level of CD3+ Ki-67+, CD4+ Ki-67+, CD8+ Ki-67+, CXCR4+ Ki-67+, CXCR7+ Ki-67+, CD45R+ Ki-67+, HLA-DR+ Ki-67+, CXCR4+ GATA3+, and GATA3+ Ki-67+ compared to typically developing individuals (10). Although previous studies have explored the relationship between immune cells and ASD, they have predominantly focused on a few specific immune cells. In addition, since current reports are mainly cross-sectional studies, the causal relationship between immune cells and ASD remains unclear. Therefore, novel and comprehensive methods are necessary to evaluate the impact of different immune cell phenotypes on ASD.

Mendelian randomization (MR) is a research method that uses genetic variation as instrumental variables to evaluate causal effects (11). Compared to traditional epidemiological research methods, MR has the advantage of being less susceptible to reverse causation and confounding variables (12). In this study, we employed MR analysis to investigate the causal effects of 731 immune cell phenotypes on the genetic susceptibility to ASD, aiming to elucidate the immune cells that may be involved in the pathogenesis of ASD.

## 2 Materials and methods

### 2.1 Study design

**Figure 1:**
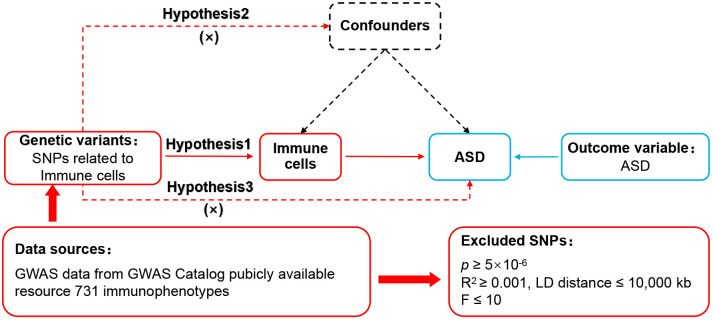
MR design for immune cell phenotypes on genetic susceptibility to ASD. MR, Mendelian randomization; ASD, autism spectrum disorder.

The framework of MR was predicated upon three fundamental assumptions (13), as illustrated in **Figure 1**. The association assumption necessitated that the single nucleotide polymorphisms (SNPs) were closely associated with the exposure. The independence assumption required that the SNPs were independent of confounding variables. The exclusivity assumption mandated that the SNPs only acted on the outcome through the exposure and not other pathways.

### 2.2 Data sources

Data on immune cell phenotypes numbered from GCST0001391 to GCST0002121 were obtained in the European Bioinformatics Institute (www.ebi.ac.uk/) (14). The datasets had 731 immune cell phenotypes containing Treg cells, mature T cells, B cells, natural killer cells, monocytes, and myeloid cells. The datasets of ASD numbered ieu-a-1185, which contained genetic information on 46,351 Europeans, were accessed in the IEU Open GWAS project (gwas.mrcieu.ac.uk/). As the database is open access, additional ethical approvals were not required.

### 2.3 Genetic instrument selection

First, SNPs closely correlated with each phenotype were identified within the immune cell phenotypes datasets, with a significance threshold set at *p* < 5×10^−6^ to fulfill the association assumption. Second, independent SNPs were searched to exclude the interference of linkage disequilibrium with the restriction of R^2^ < 0.001 and kb = 10,000. Third, SNPs demonstrating strong correlations were identified by setting F > 10 to eliminate the influence of weakly associated variables. The calculation of F was defined as F = [R^2^/(1 − R^2^)]*[(N – K − 1)/K], where K represented the number of paired samples, N denoted the total sample size, and R^2^ signified the cumulative explained variance. Fourth, SNPs with confounding variables were excluded through PhenoScanner and Google Scholar to meet the independence assumption. Fifth, mismatched SNPs were excluded based on the effect allele frequency when adjusting the allele direction of exposure and outcome. Lastly, the MR-pleiotropy residual sum and outlier method (MR-PRESSO) was employed to eliminate significantly biased SNPs (*p* < 1) to ensure the accuracy of causal inference.

### 2.4 Data analysis

The STROBE-MR was used as a guiding method (15). R 4.3.1 with the TwoSampleMR (0.5.7) package installed was used to execute the MR analysis procedures. Since it enabled unbiased causal analysis without pleiotropy, inverse variance weighted (IVW) was set as the primary evaluation tool. Weighted median, which was sensitive to outliers, and MR-Egger, which analyzed pleiotropic data, were set as secondary evaluation tools. MR-Egger was also used to analyze horizontal pleiotropy, which was required to meet the exclusivity assumption (*p* ≥ 0.05). Cochran’s Q and leave-one-out methods were used to analyze heterogeneity and sensitivity, respectively. When *p* ≥ 0.05, there was no heterogeneity in the results, and the combined effect size was robust when the significant change was not observed.

## 3 Results

### 3.1 Two-sample MR analysis

**Figure 2:**
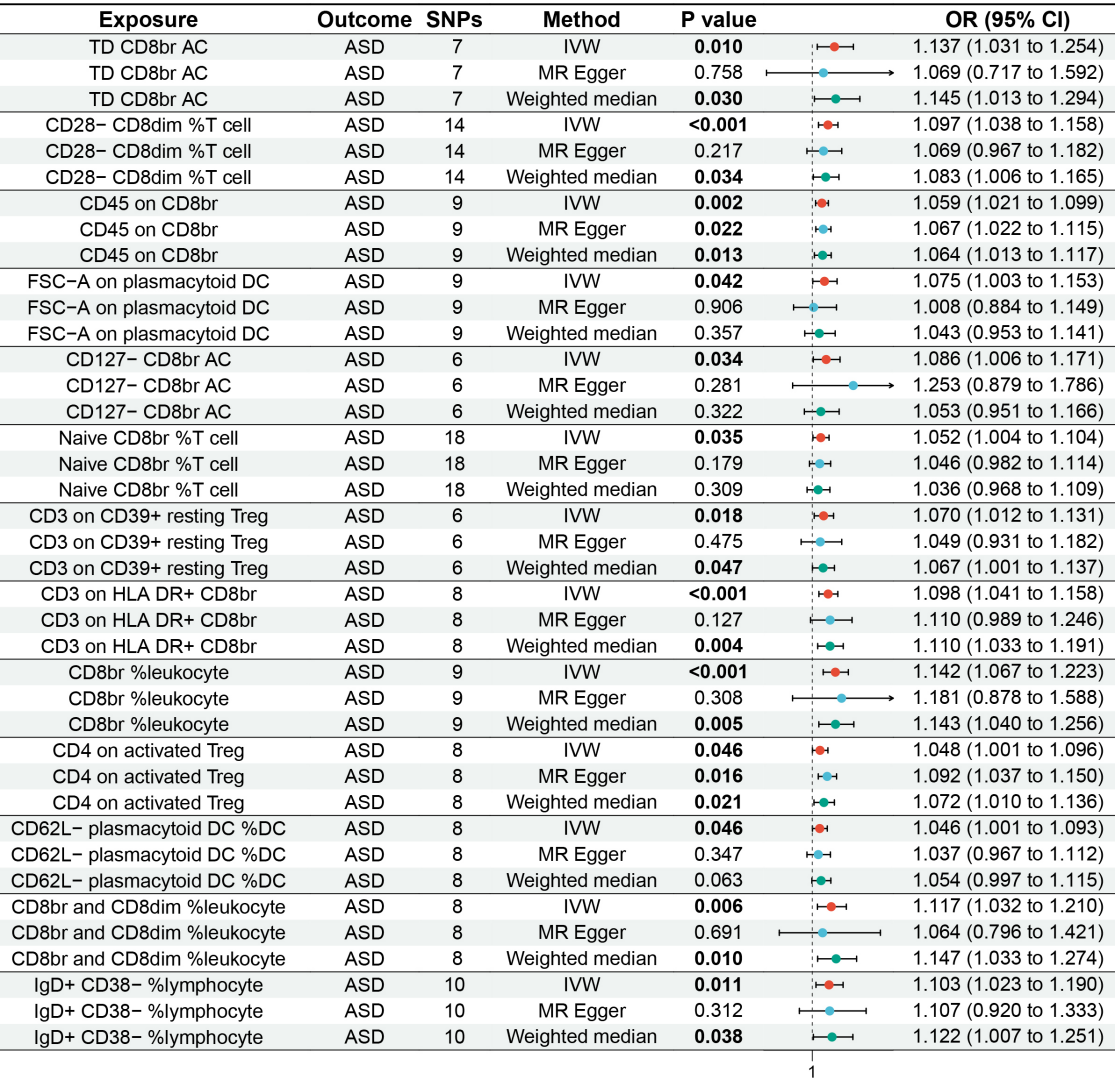
Forest plot of MR analysis for immune cell phenotypes on genetic susceptibility to ASD. MR, Mendelian randomization; ASD, autism spectrum disorder.

**Figure 3:**
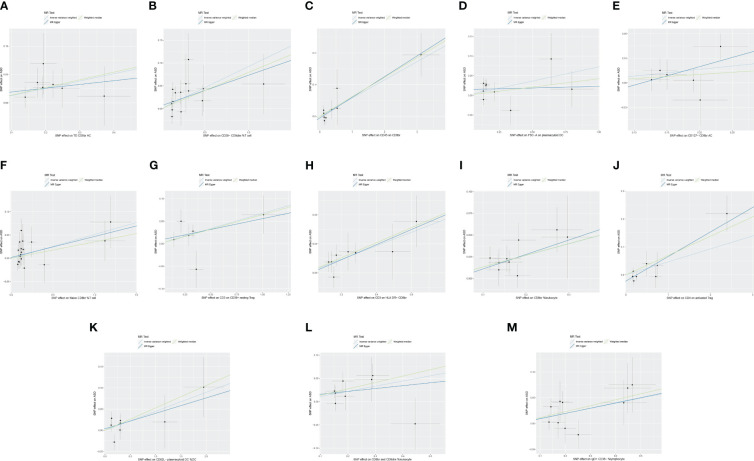
Scatter plot of MR analysis for immune cell phenotypes on genetic susceptibility to ASD. **(A)** TD CD8br AC on ASD; **(B)** CD28− CD8dim %T cell on ASD; **(C)** CD45 on CD8br on ASD; **(D)** FSC-A on plasmacytoid DC on ASD; **(E)** CD127− CD8br AC on ASD; **(F)** naive CD8br %T cell on ASD; **(G)** CD3 on CD39+ resting Treg on ASD; **(H)** CD3 on HLA DR+ CD8br on ASD; **(I)** CD8br %leukocyte on ASD; **(J)** CD4 on activated Treg on ASD; **(K)** CD62L− plasmacytoid DC %DC on ASD; **(L)** CD8br and CD8dim %leukocyte on ASD; **(M)** IgD+ CD38− %lymphocyte on ASD. MR, Mendelian randomization; ASD, autism spectrum disorder.

The MR analysis reported that 13 immune cell phenotypes were associated with increased genetic susceptibility to ASD, as shown in **Supplementary Table S1**. IVW showed that TD CD8br AC [odds ratio (OR), 1.137; 95% confidence interval (CI), 1.031–1.254; *p* = 0.010], CD28− CD8dim %T cell (OR, 1.097; 95% CI, 1.038–1.158; *p* < 0.001), CD45 on CD8br (OR, 1.059; 95% CI, 1.021–1.099; *p* = 0.002), FSC-A on plasmacytoid DC (OR, 1.075; 95% CI, 1.003–1.153; *p* = 0.042), CD127− CD8br AC (OR, 1.086; 95% CI, 1.006–1.171; *p* = 0.034), naive CD8br %T cell (OR, 1.052; 95% CI, 1.004–1.104; *p* = 0.035), CD3 on CD39+ resting Treg (OR, 1.070; 95% CI, 1.012–1.131; *p* = 0.018), CD3 on HLA DR+ CD8br (OR, 1.098; 95% CI, 1.041–1.158; *p* < 0.001), CD8br %leukocyte (OR, 1.142; 95% CI, 1.067–1.223, *p* < 0.001), CD4 on activated Treg (OR, 1.048; 95% CI, 1.001–1.096; *p* = 0.046), CD62L− plasmacytoid DC %DC (OR, 1.046; 95% CI, 1.001–1.093; *p* = 0.046), and CD8br and CD8dim %leukocyte (OR, 1.117; 95% CI, 1.032–1.210; *p* = 0.006), and IgD+ CD38− %lymphocyte (OR, 1.103; 95% CI, 1.023–1.190; *p* = 0.011) were associated with increased genetic susceptibility to ASD. The forest and scatter plots are shown in **Figures 2**, **3**, respectively. MR-Egger showed no significant horizontal pleiotropy in these results (*p* ≥ 0.05), as seen in **Supplementary Table S2**.

### 3.2 Heterogeneity and sensitivity analysis

**Figure 4:**
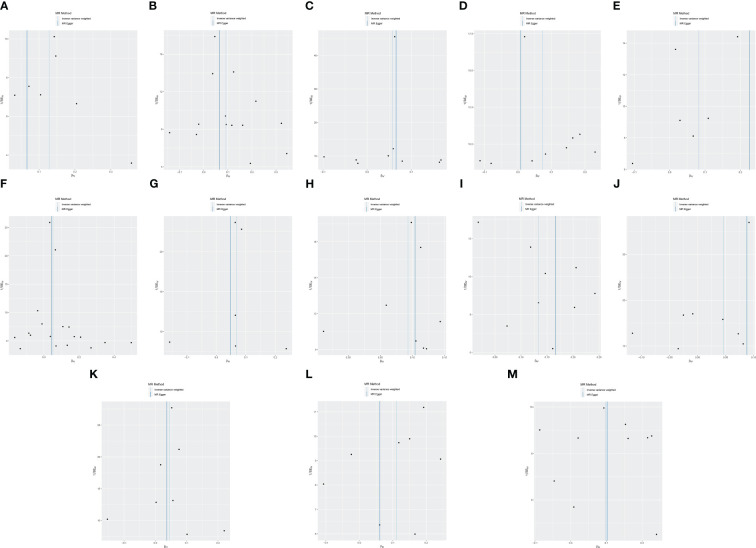
Funnel plot of MR analysis for immune cell phenotypes on genetic susceptibility to ASD. **(A)** TD CD8br AC on ASD; **(B)** CD28- CD8dim %T cell on ASD; **(C)** CD45 on CD8br on ASD; **(D)** FSC-A on plasmacytoid DC on ASD; **(E)** CD127− CD8br AC on ASD; **(F)** naive CD8br %T cell on ASD; **(G)** CD3 on CD39+ resting Treg on ASD; **(H)** CD3 on HLA DR+ CD8br on ASD; **(I)** CD8br %leukocyte on ASD; **(J)** CD4 on activated Treg on ASD; **(K)** CD62L− plasmacytoid DC %DC on ASD; **(L)** CD8br and CD8dim %leukocyte on ASD; **(M)** IgD+ CD38− %lymphocyte on ASD. MR, Mendelian randomization; ASD, autism spectrum disorder.

**Figure 5:**
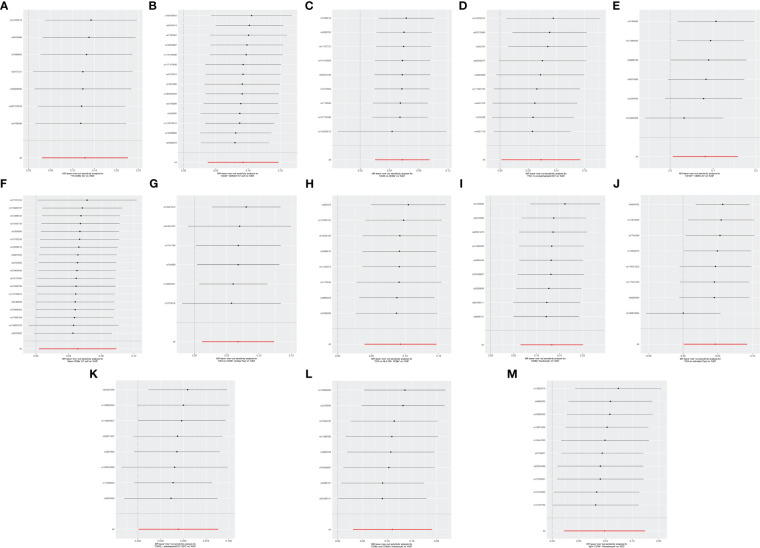
Leave-one-out sensitive analysis for immune cell phenotypes on genetic susceptibility to ASD. **(A)** TD CD8br AC on ASD; **(B)** CD28− CD8dim %T cell on ASD; **(C)** CD45 on CD8br on ASD; **(D)** FSC-A on plasmacytoid DC on ASD; **(E)** CD127− CD8br AC on ASD; **(F)** naive CD8br %T cell on ASD; **(G)** CD3 on CD39+ resting Treg on ASD; **(H)** CD3 on HLA DR+ CD8br on ASD; **(I)** CD8br %leukocyte on ASD; **(J)** CD4 on activated Treg on ASD; **(K)** CD62L− plasmacytoid DC %DC on ASD; **(L)** CD8br and CD8dim %leukocyte on ASD; **(M)** IgD+ CD38- %lymphocyte on ASD. MR, Mendelian randomization; ASD, autism spectrum disorder.

Cochran’s Q revealed no heterogeneity in the MR analysis results (*p* ≥ 0.05), as depicted in **Supplementary Table S3** and **Figure 4**. Sensitivity analysis indicated that the MR analysis results were robust, as shown in **Figure 5**.

## 4 Discussion

ASD is a persistent and disabling neurodevelopmental disorder and one of the common disorder that threatens children’s health (16). Previous views suggested that changes in immune cell subsets may be related to the occurrence or progression of ASD (17). However, the role of different immune cells in ASD is unclear due to the lack of sufficient experimental basis and clinical evidence. To our knowledge, this is the first MR analysis using large-scale GWAS data as a genetic tool to assess the causal effects of 731 immune cell phenotypes on ASD. The findings indicated that TD CD8br AC, CD8br %leukocyte, CD8br and CD8dim %leukocyte, naive CD8br %T cell, CD28− CD8dim %T cell, CD127− CD8br AC, CD45 on CD8br, CD3 on HLA DR+ CD8br, CD4 on activated Treg, CD3 on CD39+ resting Treg, IgD+ CD38− %lymphocyte, CD62L− plasmacytoid DC %DC, and FSC-A on plasmacytoid DC were associated with increased genetic susceptibility to ASD. These results are deemed credible due to the absence of pleiotropy and heterogeneity.

This MR analysis revealed that TD CD8br AC, CD8br %leukocyte, CD8br and CD8dim %leukocyte, naive CD8br %T cell, CD28− CD8dim %T cell, and CD127− CD8br AC were associated with an increased risk of ASD. TD CD8br AC refers to the absolute count of T lymphocytes with high expression of CD8. CD8br %leukocyte refers to the proportion of cells with high CD8 expression in leukocytes. CD8br and CD8dim %leukocyte refers to the proportion of cells with high CD8 and low CD8 expression in leukocytes. Naive CD8br %T cell represents the proportion of naive T cells with high expression of CD8 among all T cells. CD28− CD8dim %T cells refers to the proportion of T cells with low CD8 expression and no CD28 expression. CD127− CD8br AC refers to the absolute count of cells with high CD8 expression and no CD127 expression. These immune cell phenotypes all use CD8 as the primary marker, indicating that CD8+ T cells may play a pivotal role in the pathogenesis of ASD. CD8+ T cells primarily exert cellular immunity through cytotoxic mechanisms (18). Previous animal experiments have shown that compared to C57BL/6 (B6) mice, BTBR T+ Itpr3tf/J (BTBR) mice, a widely used animal model for autism research, exhibit higher levels of CD8+ T cells in both thymocytes and blood (19, 20). A clinical study in Spain indicated that ASD patients have a higher percentage of CD8+ T cells compared to healthy individuals (21.68% vs. 16.48%) (21). Research in Saudi Arabia has shown that compared with the general population with typical development, the numbers of CD8+ IL-16+, CD8+ TIM-3+, and CD8+ Ki-67+ cells are significantly increased in ASD patients (10, 22, 23). Another retrospective study from Beth Israel Deaconess Medical Center reported increased CD8+T and CD3+T cells infiltration around brain vessels in ASD patients (24). The study also found that the cerebrospinal fluid–glial cell barrier in ASD patients was disrupted and was thought to be related to the increase in cytotoxic T lymphocytes (24). These findings suggest an association between CD8+ T cells and ASD, indicating that TD CD8br AC, CD8br %leukocyte, CD8br and CD8dim %leukocyte, naive CD8br %T cell, CD28− CD8dim %T cell, and CD127− CD8br AC may be potential risk factors for ASD.

CD45 on CD8br refers to CD45 expression with high CD8 expression on the surface of cells. As a receptor-linked protein tyrosine phosphatase, CD45 plays a crucial role in regulating the activation process of T and B cells’ antigen receptors (25, 26). A study in Saudi Arabia revealed that compared with the general population, children with ASD have significantly increased numbers of CD45+ GM− CSF+, CD45+ IFN-γ+, CD45+ IL-6+, CD45+ IL-9+, CD45+ T-bet+, and CD45+ pStat3+ cells (27). Another study from Saudi Arabia indicated that the number of CD45R+ Ki-67+ cells in children with ASD was significantly higher than that in the general population (10). These findings support the association between CD45 and ASD, pointing to CD45 on CD8br as a potential risk factor for ASD.

CD3 on HLA DR+ CD8br means that CD3 is expressed on the surface of cells with HLA DR expression and high CD8 expression. CD3 promotes T-cell activation by tightly binding to T-cell receptors (28). Two studies on Saudi children with ASD showed a significant increase in the number of CD3+TIM-3+ and CD3+Ki-67+ cells in ASD patients compared to typically developing individuals (23). A retrospective study at Beth Israel Deaconess Medical Center also found that in addition to CD8 + T lymphocytes, CD3 + T lymphocytes also dominate the brain tissue of ASD patients (24). HLA DR is one of the human leukocyte antigen class II molecules involved in antigen presentation and immune response (29). A clinical study in Saudi Arabia indicated that children with ASD had significantly increased numbers of HLA-DR+ CD4+, HLA-DR+ CD8+, HLA-DR+ CD28+, HLA-DR+ CXCR4+, and HLA-DR+ CCR7+ cells compared to the general population (30). This study also observed higher HLA-DR+ IFN-γ+, HLA-DR+ IL-21+, and HLA-DR mRNA expression in children with ASD than in the general population (30). Alhosaini K et al. (10) also reported that the number of HLA-DR+ Ki-67+ cells in children with ASD is higher than that in the general population. These pieces of evidence support the association of CD3 and HLA DR with an increased risk of ASD, pointing to the possibility that CD3 on HLA DR + CD8br is a risk factor for ASD.

CD4 on activated Tregs and CD3 on CD39+ resting Tregs are also associated with an increased risk of ASD. CD4 on activated Treg refers to the expression of CD4 on the surface of activated regulatory T cells. CD4+ T cells regulate the activities of other immune cells by secreting different cytokines (31). Animal experiments by Uddin MN et al. (19) demonstrated that BTBR mice had higher levels of CD4+ cells in the blood and spleen compared to B6 mice. A clinical study in Saudi Arabia showed that ASD patients had higher numbers of CD4+ IL-16+, CD4+ TIM-3+, and CD4+ Ki-67+ cells compared to typically developing individuals (10, 22, 24). These findings support the association of CD4+ T cells with ASD. Considering that CD4 on activated Treg is one of the most common subtypes of CD4 T+, it may be involved in the pathogenesis of ASD. CD3 on CD39+ resting Treg refers to the expression of CD3 on the surface of CD39+ resting Treg. The relationship between CD3 and ASD has been discussed above, and CD39 is considered to be a critical factor in regulating immune system balance (32). CD39 has been confirmed to be related to tumors, inflammatory bowel disease, diabetes, and other diseases (33, 34), but there are no reports related to ASD. More research is needed in the future to explore the role of CD39 and CD3 on CD39+ resting Tregs in ASD.

In addition to the immune cell phenotypes described above, our study also revealed that IgD+ CD38− %lymphocyte, CD62L− plasmacytoid DC %DC, and FSC-A on plasmacytoid DC were associated with increased genetic susceptibility to ASD. IgD+ CD38− %lymphocyte is the percentage of lymphocytes that expresses IgD but does not express CD38. CD62L− plasmacytoid DC %DC refers to the proportion of plasmacytoid dendritic cells that do not express CD62L among dendritic cells. FSC-A on plasmacytoid DC refers to the scattering parameter of plasmacytoid dendritic cells. However, there is currently insufficient literature to support the potential connection between IgD+ CD38− %lymphocyte, CD62L− plasmacytoid DC %DC, and FSC-A on plasmacytoid DC with ASD. These associations need to be further elucidated in future research.

Although this MR analysis has enriched the genetic evidence for a causal relationship between immune cell phenotypes and ASD, some limitations remain. First, all data in this study were derived from Europeans; thus, the results only explained the causal effects of immune cell phenotypes on ASD in Europeans. Second, due to the lack of detailed baseline data, this study was unable to perform subgroup analysis based on demographic characteristics or ASD subtypes. Therefore, the applicability of these results to specific populations and ASD subtypes remains unclear. Third, there may be unidentified confounding variables between exposure and outcome, potentially increasing the bias risk of the study results. Fourth, while this MR analysis examined the link between immune cell phenotypes and ASD, it is essential to consider biological mechanisms when interpreting MR results, as relying solely on statistical effect sizes may not yield comprehensive insights. Therefore, we anticipate the following improvements in the future: first, we should keep enriching the GWAS databases to facilitate efforts toward conducting MR studies across different ethnicities and promoting health equity; second, multi-center, large-sample, and stratified clinical studies should be continued to investigate the causal effects between immune cell phenotypes and ASD patients of different races; and third, animal models should be used to explore the causal relationship between 13 immune cell phenotypes and ASD to provide more powerful biological evidence.

## 5 Conclusion

The MR analysis indicated that TD CD8br AC, CD8br %leukocyte, CD8br and CD8dim %leukocyte, naive CD8br %T cell, CD28− CD8dim %T cell, CD127− CD8br AC, CD45 on CD8br, CD3 on HLA DR+ CD8br, CD4 on activated Treg, CD3 on CD39+ resting Treg, IgD+ CD38− %lymphocyte, CD62L− plasmacytoid DC %DC, and FSC-A on plasmacytoid DC were associated with increased genetic susceptibility to ASD, including CD8^+^ T cells, Tregs, B cells, and dendritic cells. This finding emphasizes the importance of CD8 T cells and Tregs in ASD and provides a new direction for understanding the pathogenesis and drug research of ASD. However, limited by the amount of evidence, more biological research is needed in the future.

## Data availability statement

The original contributions presented in the study are included in the article/**Supplementary Material**. Further inquiries can be directed to the corresponding author.

## Author contributions

YFY: Writing – original draft, Supervision, Conceptualization. XY: Writing – original draft, Methodology, Data curation. GH: Writing – original draft, Formal analysis. YMY: Writing – original draft, Formal analysis. RY: Writing – review & editing, Supervision, Conceptualization.
