# Supplementary material for: Causal effects of 731 immune cell phenotypes on autism spectrum disorder: a Mendelian randomization study
Source: Front Psychiatry. 2024 May 16;15:1397006. doi: 10.3389/fpsyt.2024.1397006 (PMC11140572; doi:10.3389/fpsyt.2024.1397006)
Supplement: Supplementary file 1 [file Table_1.docx]

**Supplementary Table S1.1** Information of identified SNPs in exposure (TD CD8br AC) and outcomes (ASD).

|  |  | | | **Exposure (TD CD8br AC)** | | |  | **Outcome (ASD)** | | | | |
| --- | --- | --- | --- | --- | --- | --- | --- | --- | --- | --- | --- | --- |
|  | **SNP** | **EA** | **OA** | **β** | **SE** | ***p* value** |  | **Case** | **Control** | **β** | **SE** | ***p* value** |
| 1 | rs116699251 | A | G | -0.2732 | 0.05861 | 3.24E-06 |  | 18,382 | 27,969 | 0.0629504 | 0.0610527 | 0.302503 |
| 2 | rs16936840 | T | C | 0.1881 | 0.04049 | 3.50E-06 |  | 18,382 | 27,969 | 0.0562421 | 0.0375725 | 0.134421 |
| 3 | rs2637364 | C | T | -0.1164 | 0.02513 | 3.75E-06 |  | 18,382 | 27,969 | -0.0394033 | 0.0276116 | 0.153565 |
| 4 | rs36123742 | T | A | 0.1193 | 0.02587 | 4.11E-06 |  | 18,382 | 27,969 | 0.0202964 | 0.028259 | 0.472617 |
| 5 | rs38182 | T | C | -0.1541 | 0.03305 | 3.21E-06 |  | 18,382 | 27,969 | -0.083891 | 0.0630576 | 0.183391 |
| 6 | rs57641238 | C | G | -0.1546 | 0.02937 | 1.48E-07 |  | 18,382 | 27,969 | -0.028639 | 0.039451 | 0.467876 |
| 7 | rs7773849 | G | A | 1.508 | 0.2808 | 8.31E-08 |  | 18,382 | 27,969 | 0.0993091 | 0.0453545 | 0.0285516 |

SNP, single nucleotide polymorphism; EA, effect allele; OA, other allele; SE, standard error; ASD, autism spectrum disorder.

**Supplementary Table S1.2** Information of identified SNPs in exposure (CD28- CD8dim %T cell) and outcomes (ASD).

|  |  | | | **Exposure (CD28- CD8dim %T cell)** | | |  | **Outcome (ASD)** | | | | |
| --- | --- | --- | --- | --- | --- | --- | --- | --- | --- | --- | --- | --- |
|  | **SNP** | **EA** | **OA** | **β** | **SE** | ***p* value** |  | **Case** | **Control** | **β** | **SE** | ***p* value** |
| 1 | rs113075410 | G | A | 0.3121 | 0.05748 | 6.06E-08 |  | 18,382 | 27,969 | 0.107496 | 0.0577 | 0.0626095 |
| 2 | rs115125396 | C | T | 0.2263 | 0.04608 | 9.51E-07 |  | 18,382 | 27,969 | -0.00689617 | 0.0304 | 0.8194 |
| 3 | rs117147408 | G | A | 0.4819 | 0.07946 | 1.47E-09 |  | 18,382 | 27,969 | 0.0437011 | 0.0512 | 0.3931 |
| 4 | rs12986962 | G | A | -0.1604 | 0.02829 | 1.54E-08 |  | 18,382 | 27,969 | -0.0347009 | 0.0146 | 0.0172699 |
| 5 | rs13054887 | C | T | -0.1247 | 0.02667 | 3.01E-06 |  | 18,382 | 27,969 | 0.00260339 | 0.0146 | 0.8562 |
| 6 | rs1561856 | T | C | 0.1339 | 0.02919 | 4.68E-06 |  | 18,382 | 27,969 | 0.0155977 | 0.0159 | 0.3266 |
| 7 | rs182663933 | C | T | 0.2818 | 0.05987 | 2.61E-06 |  | 18,382 | 27,969 | 0.0544984 | 0.0646 | 0.399 |
| 8 | rs2274015 | T | C | 0.1183 | 0.02572 | 4.40E-06 |  | 18,382 | 27,969 | 0.0109993 | 0.0139 | 0.4295 |
| 9 | rs4796089 | C | T | -0.2225 | 0.0377 | 3.91E-09 |  | 18,382 | 27,969 | -0.0358979 | 0.0264 | 0.1729 |
| 10 | rs4869279 | A | G | -0.1312 | 0.02663 | 8.81E-07 |  | 18,382 | 27,969 | -0.0423023 | 0.0152 | 0.00528701 |
| 11 | rs626030 | G | T | 0.3132 | 0.06634 | 2.45E-06 |  | 18,382 | 27,969 | 0.0388032 | 0.0222 | 0.0799006 |
| 12 | rs7152367 | A | G | 0.4705 | 0.09932 | 2.25E-06 |  | 18,382 | 27,969 | 0.0173978 | 0.0338 | 0.6078 |
| 13 | rs75436543 | T | C | -1.173 | 0.2469 | 2.11E-06 |  | 18,382 | 27,969 | -0.0540022 | 0.0656 | 0.4109 |
| 14 | rs9457418 | T | C | 0.1229 | 0.02629 | 3.07E-06 |  | 18,382 | 27,969 | -0.0174005 | 0.0161 | 0.2801 |

SNP, single nucleotide polymorphism; EA, effect allele; OA, other allele; SE, standard error; ASD, autism spectrum disorder.

**Supplementary Table S1.3** Information of identified SNPs in exposure (CD45 on CD8br) and outcomes (ASD).

|  |  | | | **Exposure (CD45 on CD8br)** | | |  | **Outcome (ASD)** | | | | |
| --- | --- | --- | --- | --- | --- | --- | --- | --- | --- | --- | --- | --- |
|  | **SNP** | **EA** | **OA** | **β** | **SE** | ***p* value** |  | **Case** | **Control** | **β** | **SE** | ***p* value** |
| 1 | rs1015952 | T | C | 0.1213 | 0.02581 | 2.74E-06 |  | 18,382 | 27,969 | 0.00960374 | 0.0143 | 0.4999 |
| 2 | rs116030815 | C | T | -3.125 | 0.5674 | 3.93E-08 |  | 18,382 | 27,969 | -0.194201 | 0.0685 | 0.00458902 |
| 3 | rs11707721 | A | C | 0.1328 | 0.02816 | 2.49E-06 |  | 18,382 | 27,969 | -0.00280393 | 0.017 | 0.8689 |
| 4 | rs141418004 | A | G | -0.5347 | 0.1167 | 4.80E-06 |  | 18,382 | 27,969 | -0.0255024 | 0.0532 | 0.632 |
| 5 | rs147779398 | C | T | -0.5313 | 0.1151 | 4.09E-06 |  | 18,382 | 27,969 | -0.0885968 | 0.0603 | 0.1419 |
| 6 | rs2409763 | A | C | -0.1881 | 0.04102 | 4.69E-06 |  | 18,382 | 27,969 | 0.00469894 | 0.0213 | 0.8247 |
| 7 | rs60321540 | T | C | 0.3666 | 0.07926 | 3.90E-06 |  | 18,382 | 27,969 | 0.0214973 | 0.0301 | 0.474 |
| 8 | rs7179582 | A | G | -0.1181 | 0.02495 | 2.31E-06 |  | 18,382 | 27,969 | -0.019295 | 0.0145 | 0.1827 |
| 9 | rs7559619 | G | A | 0.1424 | 0.02589 | 4.05E-08 |  | 18,382 | 27,969 | -0.0139029 | 0.0146 | 0.3408 |

SNP, single nucleotide polymorphism; EA, effect allele; OA, other allele; SE, standard error; ASD, autism spectrum disorder.

**Supplementary Table S1.4** Information of identified SNPs in exposure (FSC-A on plasmacytoid DC) and outcomes (ASD).

|  |  | | | **Exposure (FSC-A on plasmacytoid DC)** | | |  | **Outcome (ASD)** | | | | |
| --- | --- | --- | --- | --- | --- | --- | --- | --- | --- | --- | --- | --- |
|  | **SNP** | **EA** | **OA** | **β** | **SE** | ***p* value** |  | **Case** | **Control** | **β** | **SE** | ***p* value** |
| 1 | rs117067704 | G | A | 0.6413 | 0.08914 | 7.97E-13 |  | 18,382 | 27,969 | 0.0926966 | 0.0658 | 0.1589 |
| 2 | rs142005014 | T | G | 0.7974 | 0.1638 | 1.19E-06 |  | 18,382 | 27,969 | 0.0156962 | 0.0461 | 0.733599 |
| 3 | rs242761 | C | T | 0.132 | 0.02779 | 2.15E-06 |  | 18,382 | 27,969 | -0.0106036 | 0.0152 | 0.484 |
| 4 | rs4431216 | C | T | -0.151 | 0.02872 | 1.55E-07 |  | 18,382 | 27,969 | -0.0247024 | 0.0145 | 0.088481 |
| 5 | rs4801716 | G | A | 0.1333 | 0.02857 | 3.19E-06 |  | 18,382 | 27,969 | 0.0306963 | 0.0141 | 0.0290302 |
| 6 | rs59715840 | C | T | 0.3368 | 0.06576 | 3.23E-07 |  | 18,382 | 27,969 | -0.038201 | 0.038 | 0.3139 |
| 7 | rs62045817 | T | C | -0.2143 | 0.0366 | 5.27E-09 |  | 18,382 | 27,969 | -0.00900038 | 0.0242 | 0.7106 |
| 8 | rs722926 | T | C | 0.1525 | 0.02762 | 3.66E-08 |  | 18,382 | 27,969 | 0.0281987 | 0.0143 | 0.0481005 |
| 9 | rs9293809 | C | T | -0.1314 | 0.02863 | 4.60E-06 |  | 18,382 | 27,969 | -0.0108015 | 0.0141 | 0.4449 |

SNP, single nucleotide polymorphism; EA, effect allele; OA, other allele; SE, standard error; ASD, autism spectrum disorder.

**Supplementary Table S1.5** Information of identified SNPs in exposure (CD127- CD8br AC) and outcomes (ASD).

|  |  | | | **Exposure (CD127- CD8br AC)** | | |  | **Outcome (ASD)** | | | | |
| --- | --- | --- | --- | --- | --- | --- | --- | --- | --- | --- | --- | --- |
|  | **SNP** | **EA** | **OA** | **β** | **SE** | ***p* value** |  | **Case** | **Control** | **β** | **SE** | ***p* value** |
| 1 | rs11060493 | C | A | 0.2008 | 0.04204 | 1.85E-06 |  | 18,382 | 27,969 | -0.020998 | 0.0315 | 0.5049 |
| 2 | rs2267983 | A | C | -0.1395 | 0.02671 | 1.88E-07 |  | 18,382 | 27,969 | -0.015205 | 0.0151 | 0.315 |
| 3 | rs3130685 | T | C | 0.1905 | 0.03102 | 9.17E-10 |  | 18,382 | 27,969 | 0.00320486 | 0.014 | 0.8199 |
| 4 | rs6908236 | C | A | -0.1266 | 0.02734 | 3.77E-06 |  | 18,382 | 27,969 | -0.00370313 | 0.0139 | 0.792899 |
| 5 | rs6915999 | A | G | -0.1501 | 0.03023 | 7.18E-07 |  | 18,382 | 27,969 | -0.00999983 | 0.0185 | 0.5891 |
| 6 | rs72849269 | A | G | 0.232 | 0.04249 | 5.06E-08 |  | 18,382 | 27,969 | 0.0444952 | 0.0161 | 0.00575506 |

SNP, single nucleotide polymorphism; EA, effect allele; OA, other allele; SE, standard error; ASD, autism spectrum disorder.

**Supplementary Table S1.6** Information of identified SNPs in exposure (Naive CD8br %T cell) and outcomes (ASD).

|  |  | | | **Exposure (Naive CD8br %T cell)** | | |  | **Outcome (ASD)** | | | | |
| --- | --- | --- | --- | --- | --- | --- | --- | --- | --- | --- | --- | --- |
|  | **SNP** | **EA** | **OA** | **β** | **SE** | ***p* value** |  | **Case** | **Control** | **β** | **SE** | ***p* value** |
| 1 | rs10183338 | T | G | 0.1206 | 0.02163 | 2.66E-08 |  | 18,382 | 27,969 | -0.00130085 | 0.0151 | 0.9311 |
| 2 | rs10474365 | T | C | 0.1374 | 0.02986 | 4.35E-06 |  | 18,382 | 27,969 | 0.0369007 | 0.0365 | 0.3119 |
| 3 | rs1064086 | A | G | -0.08993 | 0.01765 | 3.67E-07 |  | 18,382 | 27,969 | 0.00689617 | 0.015 | 0.6463 |
| 4 | rs112725843 | T | C | 0.1191 | 0.02489 | 1.79E-06 |  | 18,382 | 27,969 | 0.0205964 | 0.0207 | 0.3204 |
| 5 | rs114581570 | G | A | -1.192 | 0.2444 | 1.14E-06 |  | 18,382 | 27,969 | -0.0779975 | 0.0566 | 0.1679 |
| 6 | rs11652705 | G | A | -0.1202 | 0.02264 | 1.16E-07 |  | 18,382 | 27,969 | -0.0129952 | 0.016 | 0.4176 |
| 7 | rs116562707 | G | A | 0.3952 | 0.06626 | 2.71E-09 |  | 18,382 | 27,969 | -0.0139029 | 0.0383 | 0.715899 |
| 8 | rs11843149 | C | T | 0.09414 | 0.02024 | 3.42E-06 |  | 18,382 | 27,969 | -0.00809713 | 0.0148 | 0.584 |
| 9 | rs12555210 | G | A | 0.1558 | 0.0299 | 1.98E-07 |  | 18,382 | 27,969 | -0.020998 | 0.043 | 0.625801 |
| 10 | rs13386443 | G | A | 0.07817 | 0.01705 | 4.71E-06 |  | 18,382 | 27,969 | -0.0129952 | 0.014 | 0.3544 |
| 11 | rs17685199 | C | A | -0.1005 | 0.02146 | 2.93E-06 |  | 18,382 | 27,969 | -0.0350003 | 0.0214 | 0.102 |
| 12 | rs17767232 | A | G | 1.124 | 0.2385 | 2.55E-06 |  | 18,382 | 27,969 | 0.0374018 | 0.0434 | 0.3889 |
| 13 | rs4764066 | C | T | -0.1128 | 0.02462 | 4.76E-06 |  | 18,382 | 27,969 | -0.0077995 | 0.0275 | 0.7778 |
| 14 | rs6833962 | T | C | -0.1199 | 0.02335 | 2.97E-07 |  | 18,382 | 27,969 | -0.0596969 | 0.0256 | 0.0196002 |
| 15 | rs73404248 | A | G | -0.1427 | 0.02969 | 1.61E-06 |  | 18,382 | 27,969 | -0.0189993 | 0.0338 | 0.5752 |
| 16 | rs75368383 | C | T | 0.244 | 0.052 | 2.80E-06 |  | 18,382 | 27,969 | 0.0349021 | 0.0328 | 0.2878 |
| 17 | rs8067835 | G | A | 0.1064 | 0.02026 | 1.60E-07 |  | 18,382 | 27,969 | 0.00389759 | 0.0184 | 0.8321 |
| 18 | rs8180936 | T | C | 0.0837 | 0.01785 | 2.83E-06 |  | 18,382 | 27,969 | 0.017496 | 0.0148 | 0.2387 |

SNP, single nucleotide polymorphism; EA, effect allele; OA, other allele; SE, standard error; ASD, autism spectrum disorder.

**Supplementary Table S1.7** Information of identified SNPs in exposure (CD3 on CD39+ resting Treg) and outcomes (ASD).

|  |  | | | **Exposure (CD3 on CD39+ resting Treg)** | | |  | **Outcome (ASD)** | | | | |
| --- | --- | --- | --- | --- | --- | --- | --- | --- | --- | --- | --- | --- |
|  | **SNP** | **EA** | **OA** | **β** | **SE** | ***p* value** |  | **Case** | **Control** | **β** | **SE** | ***p* value** |
| 1 | rs16859347 | C | T | 0.2106 | 0.0431 | 1.08E-06 |  | 18,382 | 27,969 | 0.0499048 | 0.0268 | 0.0629796 |
| 2 | rs1723016 | C | T | 0.3265 | 0.02759 | 1.57E-31 |  | 18,382 | 27,969 | 0.0278955 | 0.0143 | 0.0513795 |
| 3 | rs61874707 | A | G | -1.009 | 0.2109 | 1.80E-06 |  | 18,382 | 27,969 | -0.0643999 | 0.0426 | 0.1303 |
| 4 | rs704859 | C | T | 0.141 | 0.02902 | 1.24E-06 |  | 18,382 | 27,969 | 0.00920221 | 0.0172 | 0.5905 |
| 5 | rs73001512 | G | A | -0.3578 | 0.06759 | 1.29E-07 |  | 18,382 | 27,969 | 0.0566973 | 0.0412 | 0.1694 |
| 6 | rs7741756 | A | G | -0.2843 | 0.06072 | 2.98E-06 |  | 18,382 | 27,969 | -0.0182964 | 0.0236 | 0.4379 |

SNP, single nucleotide polymorphism; EA, effect allele; OA, other allele; SE, standard error; ASD, autism spectrum disorder.

**Supplementary Table S1.8** Information of identified SNPs in exposure (CD3 on HLA DR+ CD8br) and outcomes (ASD).

|  |  | | | **Exposure (CD3 on HLA DR+ CD8br)** | | |  | **Outcome (ASD)** | | | | |
| --- | --- | --- | --- | --- | --- | --- | --- | --- | --- | --- | --- | --- |
|  | **SNP** | **EA** | **OA** | **β** | **SE** | ***p* value** |  | **Case** | **Control** | **β** | **SE** | ***p* value** |
| 1 | rs112025123 | C | T | -0.4918 | 0.1005 | 1.05E-06 |  | 18,382 | 27,969 | -0.0291024 | 0.0387 | 0.4524 |
| 2 | rs11233873 | C | A | 0.2377 | 0.05106 | 3.39E-06 |  | 18,382 | 27,969 | 0.0289964 | 0.0262 | 0.2686 |
| 3 | rs12032130 | T | C | 0.143 | 0.02834 | 4.79E-07 |  | 18,382 | 27,969 | 0.0150955 | 0.0147 | 0.3051 |
| 4 | rs1773542 | C | T | 0.2805 | 0.0264 | 6.52E-26 |  | 18,382 | 27,969 | 0.0275973 | 0.0143 | 0.0537601 |
| 5 | rs2036009 | A | G | 0.6276 | 0.1326 | 2.30E-06 |  | 18,382 | 27,969 | 0.0708964 | 0.0358 | 0.0473598 |
| 6 | rs2863272 | A | G | 0.1701 | 0.03312 | 2.96E-07 |  | 18,382 | 27,969 | 0.0243999 | 0.015 | 0.1028 |
| 7 | rs4668614 | A | G | 0.1269 | 0.02653 | 1.81E-06 |  | 18,382 | 27,969 | 0.0148985 | 0.0139 | 0.286 |
| 8 | rs864075 | T | C | -0.1558 | 0.03361 | 3.71E-06 |  | 18,382 | 27,969 | 0.00600195 | 0.0148 | 0.6844 |

SNP, single nucleotide polymorphism; EA, effect allele; OA, other allele; SE, standard error; ASD, autism spectrum disorder.

**Supplementary Table S1.9** Information of identified SNPs in exposure (CD8br %leukocyte) and outcomes (ASD).

|  |  | | | **Exposure (CD8br %leukocyte)** | | |  | **Outcome (ASD)** | | | | |
| --- | --- | --- | --- | --- | --- | --- | --- | --- | --- | --- | --- | --- |
|  | **SNP** | **EA** | **OA** | **β** | **SE** | ***p* value** |  | **Case** | **Control** | **β** | **SE** | ***p* value** |
| 1 | rs11080350 | T | C | -0.1371 | 0.02524 | 5.92E-08 |  | 18,382 | 27,969 | -0.0182964 | 0.0159 | 0.2489 |
| 2 | rs29001478 | A | G | 0.1606 | 0.03185 | 4.81E-07 |  | 18,382 | 27,969 | 0.0189984 | 0.0139 | 0.1724 |
| 3 | rs3130685 | T | C | 0.1801 | 0.02952 | 1.16E-09 |  | 18,382 | 27,969 | 0.00320486 | 0.014 | 0.8199 |
| 4 | rs3804348 | A | C | 0.2966 | 0.06161 | 1.54E-06 |  | 18,382 | 27,969 | 0.0477039 | 0.0478 | 0.3187 |
| 5 | rs3829858 | G | A | 0.1164 | 0.025 | 3.35E-06 |  | 18,382 | 27,969 | 0.0235962 | 0.0139 | 0.0906108 |
| 6 | rs62458311 | A | G | 0.2722 | 0.05919 | 4.39E-06 |  | 18,382 | 27,969 | 0.0560022 | 0.026 | 0.0310599 |
| 7 | rs6915999 | A | G | -0.1368 | 0.02884 | 2.17E-06 |  | 18,382 | 27,969 | -0.00999983 | 0.0185 | 0.5891 |
| 8 | rs6986151 | C | T | 0.1831 | 0.03986 | 4.53E-06 |  | 18,382 | 27,969 | 0.0442968 | 0.0201 | 0.0277402 |
| 9 | rs78326687 | A | C | -0.1555 | 0.03309 | 2.71E-06 |  | 18,382 | 27,969 | -0.0227979 | 0.0153 | 0.1363 |

SNP, single nucleotide polymorphism; EA, effect allele; OA, other allele; SE, standard error; ASD, autism spectrum disorder.

**Supplementary Table S1.10** Information of identified SNPs in exposure (CD4 on activated Treg) and outcomes (ASD).

|  |  | | | **Exposure (CD4 on activated Treg)** | | |  | **Outcome (ASD)** | | | | |
| --- | --- | --- | --- | --- | --- | --- | --- | --- | --- | --- | --- | --- |
|  | **SNP** | **EA** | **OA** | **β** | **SE** | ***p* value** |  | **Case** | **Control** | **β** | **SE** | ***p* value** |
| 1 | rs11615628 | A | G | -0.2479 | 0.03131 | 3.36E-15 |  | 18,382 | 27,969 | 0.00620074 | 0.0148 | 0.6782 |
| 2 | rs116421623 | T | G | -0.7415 | 0.1461 | 4.13E-07 |  | 18,382 | 27,969 | -0.0335051 | 0.0469 | 0.4746 |
| 3 | rs117421233 | A | G | -0.4885 | 0.1022 | 1.83E-06 |  | 18,382 | 27,969 | -0.0398017 | 0.0467 | 0.3937 |
| 4 | rs12039573 | T | C | -0.16 | 0.03193 | 5.72E-07 |  | 18,382 | 27,969 | 0.00550482 | 0.017 | 0.7471 |
| 5 | rs189810664 | T | C | 2.39 | 0.5071 | 2.55E-06 |  | 18,382 | 27,969 | 0.219497 | 0.0646 | 0.000681805 |
| 6 | rs7744589 | T | C | 0.7083 | 0.1524 | 3.51E-06 |  | 18,382 | 27,969 | -0.00599795 | 0.0416 | 0.8858 |
| 7 | rs9264532 | T | C | 0.188 | 0.02994 | 3.87E-10 |  | 18,382 | 27,969 | -0.0217958 | 0.0147 | 0.1373 |
| 8 | rs9266064 | T | C | -0.1748 | 0.0296 | 3.97E-09 |  | 18,382 | 27,969 | -0.0127003 | 0.0138 | 0.3591 |

SNP, single nucleotide polymorphism; EA, effect allele; OA, other allele; SE, standard error; ASD, autism spectrum disorder.

**Supplementary Table S1.11** Information of identified SNPs in exposure (CD62L- plasmacytoid DC %DC) and outcomes (ASD).

|  |  | | | **Exposure (CD62L- plasmacytoid DC %DC)** | | |  | **Outcome (ASD)** | | | | |
| --- | --- | --- | --- | --- | --- | --- | --- | --- | --- | --- | --- | --- |
|  | **SNP** | **EA** | **OA** | **β** | **SE** | ***p* value** |  | **Case** | **Control** | **β** | **SE** | ***p* value** |
| 1 | rs11000032 | A | G | 0.1292 | 0.02767 | 3.13E-06 |  | 18,382 | 27,969 | 0.0284028 | 0.0154 | 0.06501 |
| 2 | rs116054627 | A | G | 0.2959 | 0.063 | 2.74E-06 |  | 18,382 | 27,969 | 0.000699755 | 0.023 | 0.9762 |
| 3 | rs146839224 | A | G | 1.182 | 0.2447 | 1.42E-06 |  | 18,382 | 27,969 | 0.0198026 | 0.063 | 0.7538 |
| 4 | rs182833362 | A | G | 1.949 | 0.408 | 1.86E-06 |  | 18,382 | 27,969 | 0.101798 | 0.0703 | 0.1476 |
| 5 | rs3827963 | G | A | 0.1206 | 0.02628 | 4.62E-06 |  | 18,382 | 27,969 | 0.0122042 | 0.0154 | 0.4282 |
| 6 | rs55971447 | T | C | -0.3011 | 0.02899 | 6.70E-25 |  | 18,382 | 27,969 | -0.016902 | 0.0229 | 0.4605 |
| 7 | rs62297340 | A | C | 0.1816 | 0.03899 | 3.31E-06 |  | 18,382 | 27,969 | -0.0278029 | 0.0178 | 0.1177 |
| 8 | rs6535445 | C | T | -0.3029 | 0.02564 | 1.35E-31 |  | 18,382 | 27,969 | -0.0228959 | 0.0143 | 0.1094 |

SNP, single nucleotide polymorphism; EA, effect allele; OA, other allele; SE, standard error; ASD, autism spectrum disorder.

**Supplementary Table S1.12** Information of identified SNPs in exposure (CD8br and CD8dim %leukocyte) and outcomes (ASD).

|  |  | | | **Exposure (CD8br and CD8dim %leukocyte)** | | |  | **Outcome (ASD)** | | | | |
| --- | --- | --- | --- | --- | --- | --- | --- | --- | --- | --- | --- | --- |
|  | **SNP** | **EA** | **OA** | **β** | **SE** | ***p* value** |  | **Case** | **Control** | **β** | **SE** | ***p* value** |
| 1 | rs11080350 | T | C | -0.1548 | 0.0252 | 9.08E-10 |  | 18,382 | 27,969 | -0.0182964 | 0.0159 | 0.2489 |
| 2 | rs118063063 | T | C | 0.4447 | 0.08957 | 7.19E-07 |  | 18,382 | 27,969 | -0.0475949 | 0.0553 | 0.3901 |
| 3 | rs2275854 | G | A | 0.1546 | 0.03108 | 6.87E-07 |  | 18,382 | 27,969 | -0.00370313 | 0.0167 | 0.8269 |
| 4 | rs3804348 | A | C | 0.2864 | 0.06161 | 3.46E-06 |  | 18,382 | 27,969 | 0.0477039 | 0.0478 | 0.3187 |
| 5 | rs62458311 | A | G | 0.2907 | 0.05915 | 9.26E-07 |  | 18,382 | 27,969 | 0.0560022 | 0.026 | 0.0310599 |
| 6 | rs6986151 | C | T | 0.1822 | 0.03985 | 4.99E-06 |  | 18,382 | 27,969 | 0.0442968 | 0.0201 | 0.0277402 |
| 7 | rs73402222 | C | T | -0.1904 | 0.03241 | 4.59E-09 |  | 18,382 | 27,969 | -0.0116024 | 0.0299 | 0.698101 |
| 8 | rs78326687 | A | C | -0.1514 | 0.03309 | 4.90E-06 |  | 18,382 | 27,969 | -0.0227979 | 0.0153 | 0.1363 |

SNP, single nucleotide polymorphism; EA, effect allele; OA, other allele; SE, standard error; ASD, autism spectrum disorder.

**Supplementary Table S1.13** Information of identified SNPs in exposure (TD CD8br AC) and outcomes (ASD).

|  |  | | | **Exposure (TD CD8br AC)** | | |  | **Outcome (ASD)** | | | | |
| --- | --- | --- | --- | --- | --- | --- | --- | --- | --- | --- | --- | --- |
|  | **SNP** | **EA** | **OA** | **β** | **SE** | ***p* value** |  | **Case** | **Control** | **β** | **SE** | ***p* value** |
| 1 | rs10444793 | A | G | -0.4323 | 0.09225 | 2.89E-06 |  | 18,382 | 27,969 | -0.0401972 | 0.0434 | 0.3544 |
| 2 | rs12146653 | C | T | 0.1892 | 0.04126 | 4.66E-06 |  | 18,382 | 27,969 | 0.0408012 | 0.0218 | 0.0610295 |
| 3 | rs12197754 | C | T | -0.1427 | 0.03115 | 4.75E-06 |  | 18,382 | 27,969 | -0.0323994 | 0.0163 | 0.04652 |
| 4 | rs12829274 | G | A | -0.2515 | 0.05194 | 1.34E-06 |  | 18,382 | 27,969 | 0.0217958 | 0.0279 | 0.4348 |
| 5 | rs12874404 | G | A | 0.1714 | 0.02663 | 1.39E-10 |  | 18,382 | 27,969 | 0.00150113 | 0.0301 | 0.9611 |
| 6 | rs35088452 | A | C | -0.1352 | 0.02849 | 2.16E-06 |  | 18,382 | 27,969 | -0.00280393 | 0.0156 | 0.8589 |
| 7 | rs3730631 | C | T | -0.1789 | 0.03729 | 1.66E-06 |  | 18,382 | 27,969 | -0.043002 | 0.0396 | 0.2773 |
| 8 | rs62235484 | A | G | 0.4662 | 0.09386 | 7.14E-07 |  | 18,382 | 27,969 | 0.0749962 | 0.0539 | 0.1641 |
| 9 | rs6895553 | C | T | -0.199 | 0.04272 | 3.30E-06 |  | 18,382 | 27,969 | 0.00920221 | 0.0292 | 0.7526 |
| 10 | rs72706301 | A | G | 0.448 | 0.09406 | 1.98E-06 |  | 18,382 | 27,969 | 0.0687982 | 0.0484 | 0.1552 |

SNP, single nucleotide polymorphism; EA, effect allele; OA, other allele; SE, standard error; ASD, autism spectrum disorder.

**Supplementary Table S2** The results of MR-Egger intercept analysis.

| **Exposure** | **Outcome** | **Egger_intercept** | **SE** | ***p* value** |
| --- | --- | --- | --- | --- |
| TD CD8br AC | ASD | 0.01254979 | 0.039960756 | 0.766161377 |
| CD28- CD8dim %T cell | ASD | 0.006427428 | 0.010682117 | 0.558570701 |
| CD45 on CD8br | ASD | -0.005275331 | 0.007932027 | 0.52730397 |
| FSC-A on plasmacytoid DC | ASD | 0.014706134 | 0.012992082 | 0.294944656 |
| CD127- CD8br AC | ASD | -0.025347904 | 0.031209954 | 0.462260228 |
| Naive CD8br %T cell | ASD | 0.001912526 | 0.006453811 | 0.770781967 |
| CD3 on CD39+ resting Treg | ASD | 0.007733073 | 0.020522011 | 0.725446135 |
| CD3 on HLA DR+ CD8br | ASD | -0.002821343 | 0.013269168 | 0.838660016 |
| CD8br %leukocyte | ASD | -0.005594755 | 0.02478114 | 0.827833004 |
| CD4 on activated Treg | ASD | -0.02242902 | 0.009827824 | 0.062610799 |
| CD62L- plasmacytoid DC %DC | ASD | 0.003636047 | 0.011444701 | 0.761474185 |
| CD8br and CD8dim %leukocyte | ASD | 0.009710591 | 0.027834973 | 0.739108398 |
| IgD+ CD38- %lymphocyte | ASD | -0.00083098 | 0.019618864 | 0.967252818 |

ASD, autism spectrum disorder.

**Supplementary Table S3** The results of Cochran's Q analysis.

| **Exposure** | **Outcome** | **Method** | **Q** | **Q_df** | **Q_*p* val** |
| --- | --- | --- | --- | --- | --- |
| TD CD8br AC | ASD | MR Egger | 1.562101613 | 5 | 0.90579396 |
| TD CD8br AC | ASD | Inverse variance weighted | 1.660730818 | 6 | 0.948112473 |
| CD28- CD8dim %T cell | ASD | MR Egger | 14.24084034 | 12 | 0.285593843 |
| CD28- CD8dim %T cell | ASD | Inverse variance weighted | 14.67048963 | 13 | 0.328372491 |
| CD45 on CD8br | ASD | MR Egger | 4.513701484 | 7 | 0.719066762 |
| CD45 on CD8br | ASD | Inverse variance weighted | 4.956015922 | 8 | 0.762266712 |
| FSC-A on plasmacytoid DC | ASD | MR Egger | 8.831164025 | 7 | 0.265006209 |
| FSC-A on plasmacytoid DC | ASD | Inverse variance weighted | 10.44760336 | 8 | 0.235003918 |
| CD127- CD8br AC | ASD | MR Egger | 4.302939457 | 4 | 0.366557161 |
| CD127- CD8br AC | ASD | Inverse variance weighted | 5.012522251 | 5 | 0.414353867 |
| Naive CD8br %T cell | ASD | MR Egger | 13.5965339 | 16 | 0.628743094 |
| Naive CD8br %T cell | ASD | Inverse variance weighted | 13.68435164 | 17 | 0.689305851 |
| CD3 on CD39+ resting Treg | ASD | MR Egger | 5.602418952 | 4 | 0.230872383 |
| CD3 on CD39+ resting Treg | ASD | Inverse variance weighted | 5.801293873 | 5 | 0.326036593 |
| CD3 on HLA DR+ CD8br | ASD | MR Egger | 2.651104452 | 6 | 0.851188174 |
| CD3 on HLA DR+ CD8br | ASD | Inverse variance weighted | 2.696313394 | 7 | 0.911603895 |
| CD8br %leukocyte | ASD | MR Egger | 4.32482238 | 7 | 0.741695831 |
| CD8br %leukocyte | ASD | Inverse variance weighted | 4.375792969 | 8 | 0.821727175 |
| CD4 on activated Treg | ASD | MR Egger | 5.055597129 | 6 | 0.536702427 |
| CD4 on activated Treg | ASD | Inverse variance weighted | 10.26401482 | 7 | 0.174099134 |
| CD62L- plasmacytoid DC %DC | ASD | MR Egger | 7.368862415 | 6 | 0.28807729 |
| CD62L- plasmacytoid DC %DC | ASD | Inverse variance weighted | 7.492827494 | 7 | 0.379428415 |
| CD8br and CD8dim %leukocyte | ASD | MR Egger | 7.128651129 | 6 | 0.309113409 |
| CD8br and CD8dim %leukocyte | ASD | Inverse variance weighted | 7.27325031 | 7 | 0.400994439 |
| IgD+ CD38- %lymphocyte | ASD | MR Egger | 7.737329984 | 8 | 0.459540097 |
| IgD+ CD38- %lymphocyte | ASD | Inverse variance weighted | 7.739124031 | 9 | 0.560635017 |

ASD, autism spectrum disorder.
